# Racemose Neurocysticercosis Presenting As Vasculitic Infarct and Obstructive Hydrocephalus: A Rare Presentation

**DOI:** 10.7759/cureus.78418

**Published:** 2025-02-03

**Authors:** Rishabh Rawat, Gopinadh Tummagunta, Geeta Kampani, Hrishabh Singh, Fayaz Hussain Azad

**Affiliations:** 1 Department of Internal Medicine, Atal Bihari Vajpayee Institute of Medical Sciences (ABVIMS) and Dr. Ram Manohar Lohia Hospital, New Delhi, IND

**Keywords:** extraparenchymal neurocysticercosis, ischemic stroke, obstructive hydrocephalus, racemose neurocysticercosis, vasculitic infarct

## Abstract

Racemose neurocysticercosis (NCC) is a rare and aggressive form of parasitic infection that primarily involves the subarachnoid space. It can lead to serious complications such as hydrocephalus and ischemic stroke due to vasculitis. Early recognition of this condition is essential for preventing long-term neurological damage. This report discusses a case of a 68-year-old male who presented with sudden onset right-sided hemiplegia and facial palsy despite having no previous comorbidities. Imaging studies revealed extraparenchymal cystic lesions consistent with racemose NCC, leading to obstructive hydrocephalus and stroke.

The patient was treated with a combination of albendazole, praziquantel, and dexamethasone. Following three weeks of therapy, he demonstrated marked clinical improvement and was subsequently discharged with instructions for follow-up care. This case highlights the critical need for timely diagnosis and management of racemose NCC to improve neurological outcomes. Prompt treatment using antiparasitic agents and steroids can play a pivotal role in facilitating recovery and minimizing complications. Recognizing racemose NCC as a potential cause of stroke and hydrocephalus in endemic areas is vital for early intervention and enhanced patient outcomes.

## Introduction

Neurocysticercosis (NCC) is a central nervous system infection resulting from the larval form of the parasitic tapeworm Taenia solium. It is the most common parasitic disease of the brain and a leading cause of acquired epilepsy worldwide, particularly in developing regions where hygiene and sanitation standards are poor. Humans contract this condition by ingesting Taenia solium eggs through contaminated food or water or via direct contact with individuals harboring the adult tapeworm. After ingestion, the eggs hatch in the intestine, releasing oncospheres that penetrate the intestinal wall, enter the bloodstream, and migrate to various tissues, including the brain [1,2].

The clinical presentation of NCC varies widely, influenced by the number, size, location, and developmental stage of the cysts, as well as the host’s immune response. The manifestations range from asymptomatic cases to severe neurological deficits, including seizures, headaches, focal neurological symptoms, and signs of raised intracranial pressure. The disease can also present with psychiatric symptoms, such as cognitive impairment and personality changes, adding to its diagnostic complexity [3].

NCC is categorized into parenchymal and extraparenchymal types. Parenchymal NCC, which affects the brain's cortex, is more common and generally associated with a better prognosis. In contrast, extraparenchymal forms, which include ventricular, subarachnoid, and racemose NCC, are less common but more severe. The racemose form is particularly aggressive, characterized by the presence of multiple grape-like cystic structures in the subarachnoid space, often without an associated scolex (the head of the larva). This variant is notorious for causing severe inflammation, hydrocephalus, and other life-threatening complications [4].

Racemose NCC is a relatively rare variant of the disease. The cysts in this form tend to grow in the subarachnoid space, especially at the base of the brain and can reach considerable sizes, compressing surrounding structures. This compression can result in hydrocephalus (due to obstruction of cerebrospinal fluid pathways), cranial nerve palsies, and vascular compromise. The diagnosis of racemose NCC is challenging due to its atypical presentation and the absence of a clear scolex, which is the hallmark of typical cysticercosis [5].

Neuroimaging is essential for diagnosing NCC. Computed tomography (CT) and magnetic resonance imaging (MRI) are the primary tools used to detect cysts and their locations, the degree of inflammation, and associated complications like hydrocephalus or edema. In the case of racemose NCC, MRI often reveals large, multiloculated cysts without a scolex, commonly located in the subarachnoid cisterns, Sylvian fissure, or interhemispheric fissure. CT scans may also show calcified cysts or evidence of chronic infection, but MRI is more sensitive, particularly for detecting active or complicated lesions [6].

Serological tests, such as enzyme-linked immunosorbent assay (ELISA) and enzyme-linked immunoelectrotransfer blot (EITB), are commonly employed to detect Taenia solium-specific antibodies or antigens, aiding in diagnosis. A positive test, particularly in conjunction with neuroimaging findings, is often sufficient to confirm the diagnosis. In racemose NCC, these tests can be particularly helpful due to the often-challenging imaging diagnosis [7,8].

The treatment of NCC is multifaceted and depends on the number, location, and stage of cysts, as well as the presence of complications. Antiparasitic drugs such as albendazole and praziquantel are the mainstay of treatment, often combined with corticosteroids to reduce inflammation caused by the death of the cysts. Albendazole is typically dosed at 15 mg/kg/day and praziquantel at 50 mg/kg/day. The racemose form, due to its extensive involvement, may require prolonged courses of antiparasitic therapy and higher doses of corticosteroids to manage inflammation. In cases with significant hydrocephalus, surgical interventions, such as ventriculoperitoneal shunting, may be required to relieve intracranial pressure. Additionally, anticonvulsant therapy is often necessary in patients with seizures [9,10]. This case report presented the diagnostic findings and treatment of a 68-year-old male diagnosed with racemose NCC.

## Case presentation

A 68-year-old male with no known comorbidities presented with complaints of weakness of the right upper and lower limbs and slurring of speech for the last 12 hours. It was a sudden onset, associated with a deviation in the angle of the mouth to the left side. There was no associated history of fever, abnormal body movements, difficulty in closing any eye, or sensory loss at the time of admission. On examination, his vitals were stable, with a blood pressure of 110/70 mmHg, a pulse rate of 90 beats per minute, and oxygen saturation (SpO2) of 97% on room air. He was afebrile, and his random blood sugar was 135 mg/dL. Neurological examination revealed an increased muscle tone in the right upper and lower limbs, with muscle power graded at 1/5 in these limbs. In contrast, the left upper and lower limbs had normal tone and power (5/5). The plantar reflex was extensor on the right side and flexor on the left, with a right-sided upper motor neuron (UMN) type facial nerve palsy. Examination of other systems was unremarkable.

**Figure 1:**
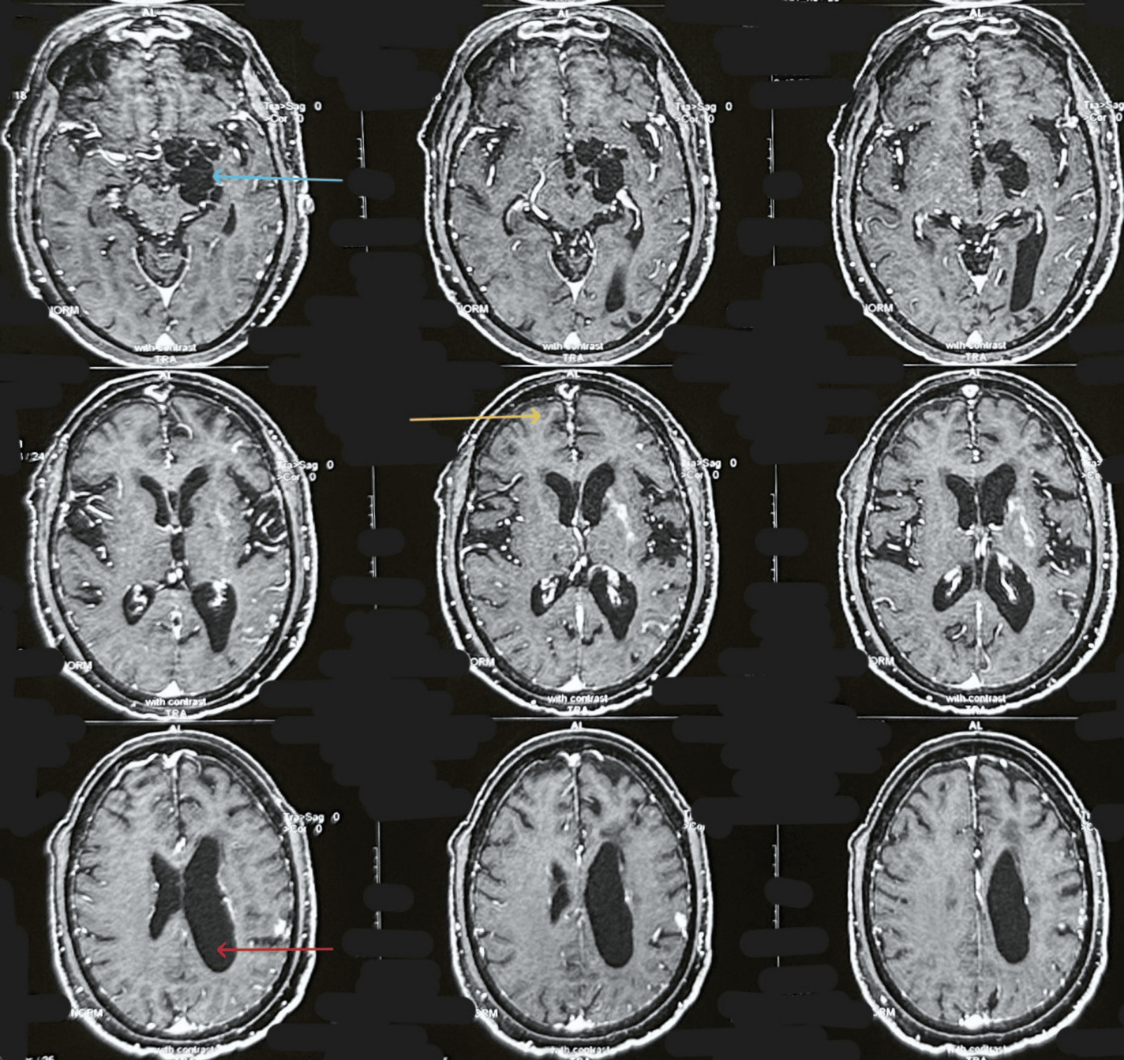
Contrast-enhanced MRI showing vesicular stage parenchymal neurocysticercosis in the right frontal lobe, racemose neurocysticercosis in the left crural cistern, and obstructive hydrocephalus of the left lateral ventricle The blue arrow indicates racemose neurocysticercosis; the yellow arrow highlights parenchymal neurocysticercosis in the vesicular stage with ring-enhancing lesions and eccentric scolex; the red arrow points to features of obstructive hydrocephalus caused by multiloculated cystic lesions.

**Figure 2:**
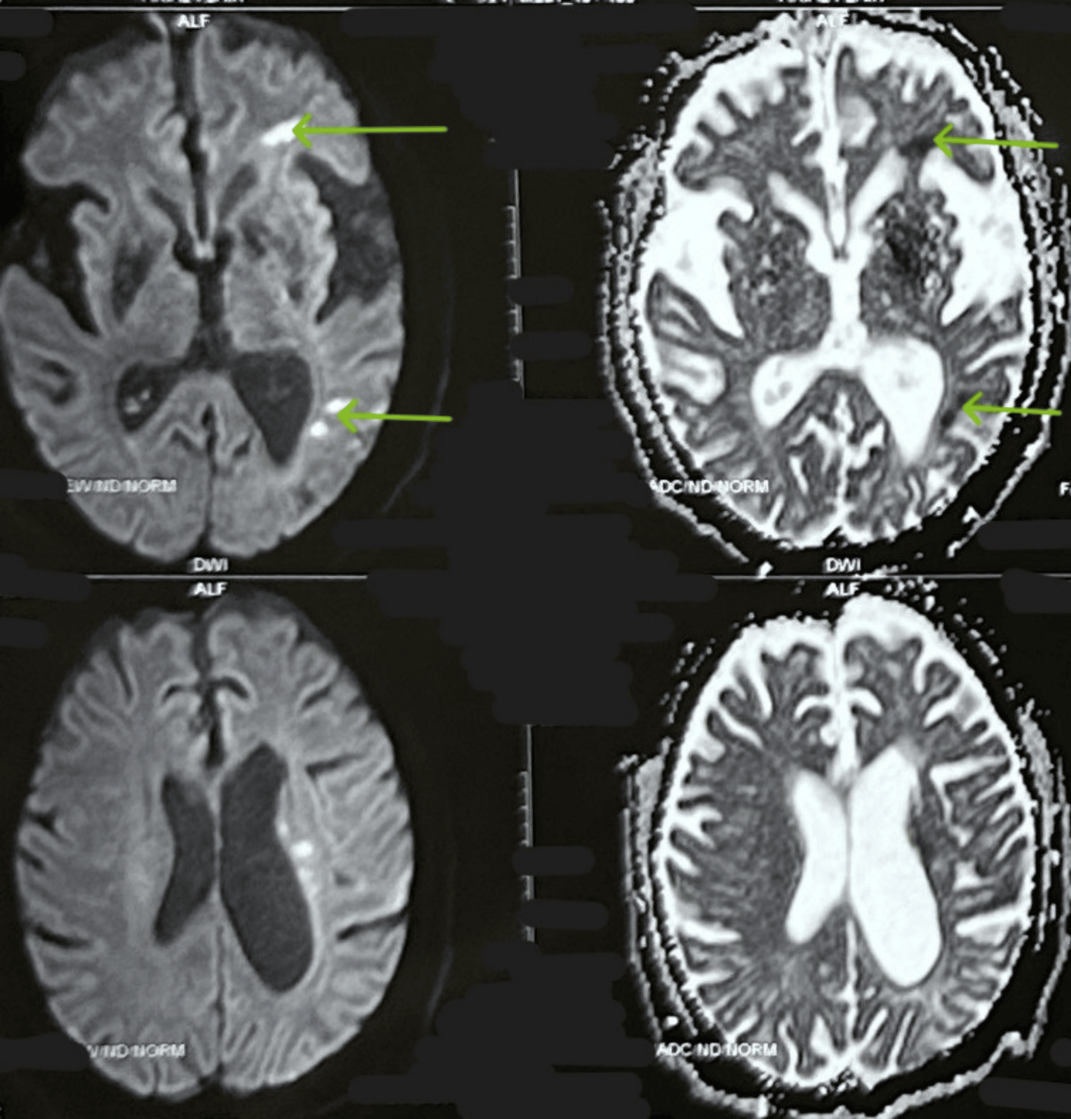
Diffusion-weighted imaging (DWI) with corresponding apparent diffusion coefficient (ADC) maps showing acute lacunar infarcts in the left frontal and occipital lobes The green arrows denote the areas of lacunar infarcts in the left frontal and occipital lobes.

The following series of investigations were conducted for the patient (Table 1, Figures 1-2).

**Table 1:** Laboratory and neuroimaging investigations of the case

Parameter	Result	Normal Reference Range
Hemoglobin	14.6 g/dL	13.0-17.0 g/dL
Total Leucocyte Count	11,000 cells/mm^3^	4,000-11,000 cells/mm^3^
Platelet Count	3.1 lakh/mm^3^	1.5-4.5 lakh/mm^3^
Blood Urea	27 mg/dL	7-20 mg/dL
Serum Creatinine	0.9 mg/dL	0.6-1.3 mg/dL
Total Bilirubin	0.7 mg/dL	0.1-1.2 mg/dL
Aspartate Transaminase	34 U/L	5-40 U/L
Alanine Transaminase	47 U/L	7-56 U/L
Alkaline Phosphatase	80 U/L	44-147 U/L
Sodium	135 mmol/L	135-145 mmol/L
Potassium	3.9 mmol/L	3.5-5.1 mmol/L
Non-contrast Computed Tomography of the Head	Cystic hypodense lesion in left mesial temporal lobe with dilated left lateral ventricle	Not applicable
Contrast-Enhanced Magnetic Resonance Imaging (CEMRI) of the Brain and MR Angiography of Head and Neck Vessels	Ring-enhancing lesions observed in the right frontal and occipital lobes, with eccentric enhancing scolex, indicative of the vesicular stage of neurocysticercosis. Additionally, a well-defined extra-axial multiloculated cystic lesion was noted in the left crural cistern and Sylvian fissure, abutting the bifurcation of the internal carotid artery into the middle cerebral and anterior cerebral arteries, as well as the posterior communicating artery. Left lateral ventricle was dilated, but remaining ventricular system was not. Findings suggestive of racemose neurocysticercosis associated with obstructive hydrocephalus. Lacunar Infarcts were present in the left frontal and occipital lobes. No abnormality in the head and neck vessels detected.	Not applicable
*Taenia solium* IgG Antibodies (Enzyme-Linked Immunosorbent Assay (ELISA))	Positive	Negative
Electrocardiogram (ECG)	Normal sinus rhythm	Normal sinus rhythm
2D Echocardiography	Normal	Normal
Glycated Hemoglobin (HbA1c)	5.6	<5.7
Serum Cholesterol, Triglycerides, Low-Density Lipoprotein (LDL)	Normal	Normal

The patient was diagnosed with racemose NCC associated with obstructive hydrocephalus and vasculitic infarcts. The patient was started on a treatment regimen consisting of oral albendazole (15 mg/kg/day), oral praziquantel (50 mg/kg/day), and oral dexamethasone (0.1 mg/kg/day). Over the course of treatment, there was significant clinical improvement, with muscle power in the right upper and lower limbs improving from 1/5 to 4/5 and resolution of the right UMN facial palsy. The patient was discharged after two weeks of hospital stay on dual anthelmintic therapy with the plan to follow up with repeat neuroimaging. On a follow-up visit after three weeks, the patient’s neurological deficit had completely recovered.

## Discussion

NCC is a major contributor to neurological disorders in endemic areas. Racemose NCC predominantly involves the subarachnoid space and basal cisterns. In this case, a 68-year-old male presented with a sudden onset of right-sided hemiplegia and UMN-type facial palsy. The absence of fever and abnormal movements directed the clinical suspicion toward a non-infectious central nervous system etiology, which was later confirmed through neuroimaging [11,12]. In the present case, the diagnosis was validated by the presence of ring-enhancing lesions with eccentric enhancing scolex in the right frontal and occipital lobes, suggestive of the vesicular stage of NCC, as well as a multiloculated cystic lesion in the left crural cistern and Sylvian fissure, characteristic of the racemose variant of NCC. The identification of Taenia soliumIgG antibodies further solidified the diagnosis [13].

Racemose NCC is known to occur in 3.6% of patients with NCC [14]. It differs from other forms of the disease due to its location in the subarachnoid space and its more aggressive course, often leading to hydrocephalus, meningeal irritation, or stroke-like symptoms. In our patient, the presentation with acute right hemiplegia and lacunar infarcts highlights the potential for ischemic events in racemose NCC, likely due to vasculitis or compression of cerebral vasculature by cystic lesions [15,16].

The treatment protocol for racemose NCC involves antiparasitic agents like albendazole and praziquantel combined with corticosteroids to mitigate the inflammatory response triggered by the death of the cysts. In this case, the patient responded well to a combination of albendazole, praziquantel, and dexamethasone. There was a marked improvement in motor power and a resolution of the UMN facial palsy, indicating a favorable response to therapy. However, the need for long-term follow-up with serial imaging is critical due to the possibility of relapse or complications like hydrocephalus or persistent seizures [17,18].

## Conclusions

This case highlights the importance of considering racemose NCC in the differential diagnosis of sudden-onset neurological deficits, particularly in endemic regions where parasitic infections are prevalent. The patient initially presented with stroke-like symptoms that could have been misinterpreted as a cerebrovascular event. However, neuroimaging and serological testing identified racemose NCC in the subarachnoid space, a rare and severe form of the disease associated with complications such as hydrocephalus and increased intracranial pressure. Prompt intervention with antiparasitic agents and corticosteroids led to significant neurological improvement. This case underscores the critical role of early diagnosis and timely treatment, along with long-term follow-up, to monitor for potential complications and ensure optimal patient outcomes. Further research is needed to better understand the pathophysiology, optimize diagnostic protocols, and develop standardized treatment guidelines for this aggressive form of NCC.
